# Metabolic dysfunction–associated steatotic liver disease and the gut microbiome: pathogenic insights and therapeutic innovations

**DOI:** 10.1172/JCI186423

**Published:** 2025-04-01

**Authors:** Bernd Schnabl, Christopher J. Damman, Rotonya M. Carr

**Affiliations:** 1 Department of Medicine, Division of Gastroenterology, UCSD, San Diego, California, USA.; 2 Department of Medicine, VA San Diego Healthcare System, San Diego, California, USA.; 3 Department of Medicine, Division of Gastroenterology, University of Washington, Seattle, Washington, USA.

## Abstract

Metabolic dysfunction–associated steatotic liver disease (MASLD) is a major cause of liver disease worldwide, and our understanding of its pathogenesis continues to evolve. MASLD progresses from steatosis to steatohepatitis, fibrosis, and cirrhosis, and this Review explores how the gut microbiome and their metabolites contribute to MASLD pathogenesis. We explore the complexity and importance of the intestinal barrier function and how disruptions of the intestinal barrier and dysbiosis work in concert to promote the onset and progression of MASLD. The Review focuses on specific bacterial, viral, and fungal communities that impact the trajectory of MASLD and how specific metabolites (including ethanol, bile acids, short chain fatty acids, and other metabolites) contribute to disease pathogenesis. Finally, we underscore how knowledge of the interaction between gut microbes and the intestinal barrier may be leveraged for MASLD microbial-based therapeutics. Here, we include a discussion of the therapeutic potential of prebiotics, probiotics, postbiotics, and microbial-derived metabolites.

## Introduction

Metabolic dysfunction–associated steatotic liver disease (MASLD) affects approximately 30% of the global population and arises from the interaction of environmental and biological risk factors (1, 2). It is a systemic metabolic disorder impacting multiple organs. Hepatic effects include hepatic steatosis (lipid accumulation in hepatocellular lipid droplet organelles), with some patients progressing to metabolic dysfunction–associated steatohepatitis (MASH). MASH is the inflammatory stage of MASLD that can progress to fibrosis, cirrhosis, and hepatocellular carcinoma (HCC). The pathogenesis of MASLD is multifactorial and includes environmental exposures, genetic predispositions, impairment in insulin signaling and lipid metabolism, augmentation of lipotoxicity and other inflammatory pathways, disruptions of the intestinal barrier function, and derangements of the intestinal microbiome (3–5).

Environmental factors include nutrients and toxins, while biological factors encompass genetic, metabolic, and microbial contributors. The intestinal, or gut, microbiome is the most studied of the microbial factors that influence MASLD pathogenesis, risk, and severity. This commensal community includes trillions of bacterial, viral, and fungal organisms that influence host nutrition, digestion, intestinal motility, metabolism, and immunity. These organisms also interact with one another to effect microbial functions, which in turn, influence host biology. The balance (or diversity) among these organisms within a single host environment (α diversity) and between host environments (β diversity) can influence both health and disease. Namely, α diversity includes the number of species and their relative abundance, where higher α diversity is typically associated with a healthy gut microbial environment. β Diversity is a measurement of how these microbial communities vary between environments, often a result of ecologic influences (6). We have also demonstrated that there are differences in abundance of these microbes along the intestinal tract, where small intestinal microbiota communities and their metabolites differed from that of the colon, and we suspect that where in the intestinal tract microbes reside has biological consequences (7).

Utilizing high-throughput genetic sequencing, in vitro and in vivo functional assays, complex bioinformatics, and multiomic platforms, researchers can gain insight into what impacts the α and β diversity in biological samples from MASLD patients. This approach identifies both the organisms affecting biodiversity and their functions in MASLD pathogenesis, potentially suggesting therapeutic targets. However, a sizable challenge persists: just as microorganisms impact the host in MASLD, liver disease influences the gut microbiome, making it difficult to determine whether gut microbiome disruptions are an initial cause or a consequence of MASLD pathogenesis.

Herein, we review the evidence of the pathogenic role of the gut microbiome in clinical MASLD, focusing on bacterial, viral, and fungal communities and their metabolites. Further, we explore how the gut microbiome can be leveraged for innovative MASLD therapies.

## The liver-gut axis and intestinal barrier function

The liver and intestine engage in bidirectional communication due to their anatomical connection and the immunologic, metabolic, and hormonal interactions between them. Anatomically, the splanchnic venous intestinal outflow connects to the liver’s portal venous inflow. Portal venous blood contains nutrients, toxins, reabsorbed bile acids, and microbial and antimicrobial products. In the opposing direction, the liver produces bile, which enters the duodenum at the ampulla of Vater and contains emulsifying bile acids, trace elements, immune factors, and other lipids and metabolites. The biologic life cycle of bile acids requires intestinal microbiota for deconjugation, and conversely, bile acids exert often toxic effects on intestinal microbiota (8). Physiologically, the liver and gut are also connected via shared hormones. For example, cholecystokinin is produced by enteroendocrine cells to elicit bile production from the liver; fibroblast growth factor-19 (FGF-19) is produced in the intestine and regulates hepatic bile acid synthesis (9); and insulin-like growth factor, produced by the liver, helps maintain intestinal epithelial integrity (10).

**Figure 1:**
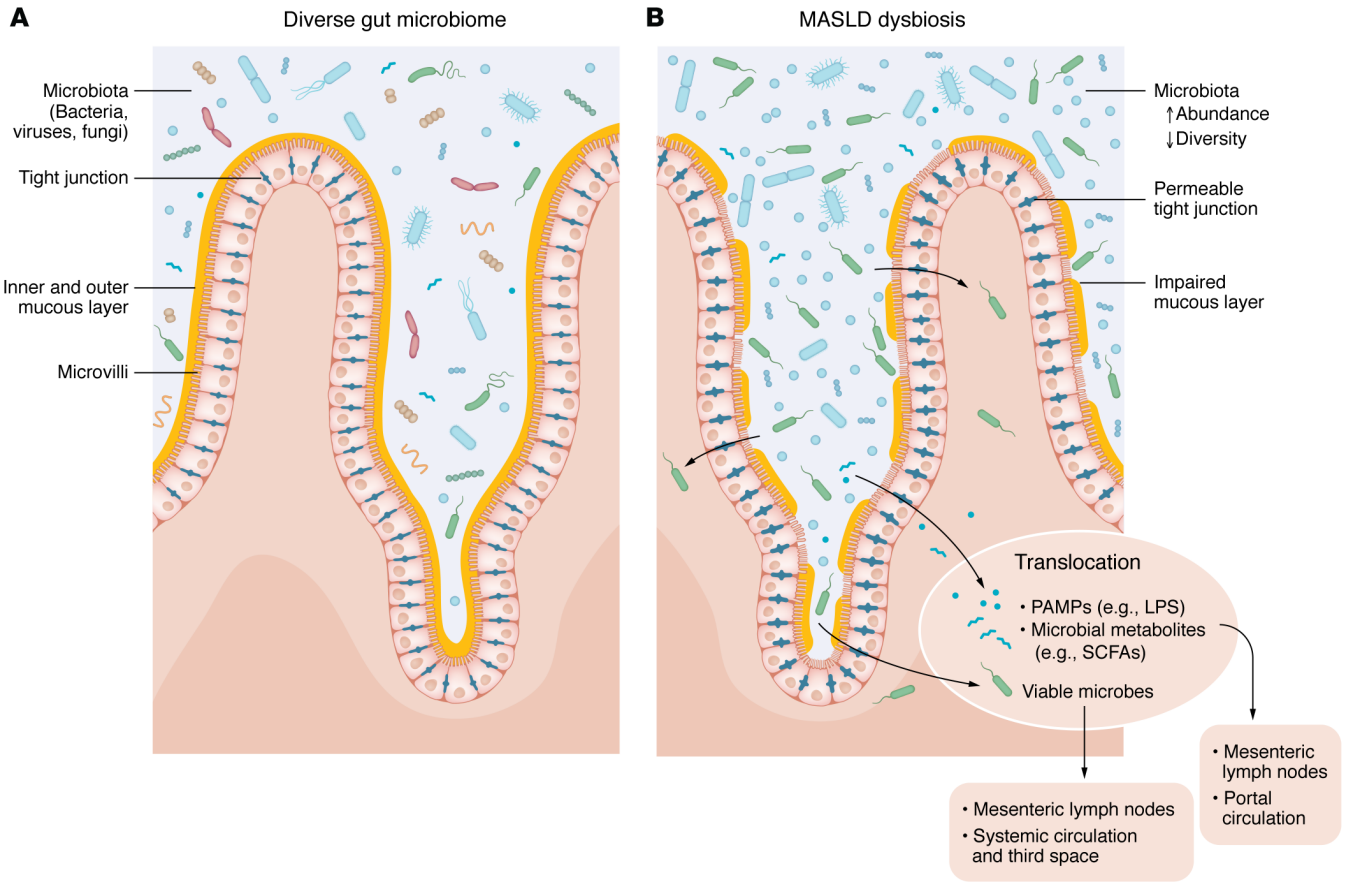
Gut barrier dysfunction in MASLD. The intestinal epithelial barrier provides the main line of defense against translocation of gut microbiota. The barrier includes epithelial cell microvilli, an outer and inner mucous layer, and tight junction proteins located between adjacent epithelial cells. The figure compares (**A**) a normal intestinal epithelial barrier with diverse gut microbiome to (**B**) an impaired intestinal epithelial barrier as observed in MASLD with subsequent translocation of bacterial products, metabolites, and viable bacteria.

This highly regulated enterohepatic circulation is fortified by the intestinal barrier that limits paracellular leakage of intestinal lumen substances directly into the systemic circulation or prevents lumen contents from reaching high concentrations in the portal circulation. The intestinal barrier has several components: epithelial cells, overlying mucous layers (a superficial, thick outer mucous layer and an inner, thin, unstirred layer), electrostatic microvilli, and intercalating proteins that create the apical junctional complex between adjacent epithelial cells (Figure 1). Working in concert with intestinal epithelial cells to fortify the barrier are Paneth cells, which secrete defensins and other antimicrobials, and the lamina propria, which is located beneath the epithelial cell basement membrane and has a role in innate and acquired immunity (11, 12).

From apical to basal distribution, there are three domains that comprise the apical junctional complex: the zonula occludens (ZO), zonula adherens, and desmosome. While the intestinal barrier and the apical junctional complex within the barrier are structurally complex, investigators often use the integrity of the ZO region, or “tight junction,” or urinary recovery of orally administered molecules whose size should preclude intestinal epithelial paracellular passage as proxies for permeability (11).

While most research on intestinal barrier function in MASLD has involved the role of the epithelial cell tight junction, less studied is the role of the gut vascular barrier (GVB). Akin to the blood-brain barrier, this microvasculature barrier provides an additional guard against translocation of bacteria and potentially hepatotoxic molecules. The GVB derives from intestinal blood endothelial cells. Like intestinal epithelial cells, they also have a tight junction and associated tight junction and adherens junction proteins. The gut endothelial cells are surrounded by enteric glial cells and pericytes organized to form a vascular unit (13). Understanding these intricate, highly regulated interfaces between the gut and the liver is crucial to comprehending how disruptions to this barrier contribute to the gut microbiome’s influence on MASLD pathogenesis.

## Role of gut barrier dysfunction in MASLD pathogenesis

Studies on humans with MASLD indicate a compromised intestinal barrier. A metaanalysis of 14 studies involving adult and pediatric MASLD patients diagnosed via liver biopsy or ultrasound showed increased intestinal permeability, measured by urinary recovery of orally administered sugars or ZO levels, compared with healthy controls (14). Preclinical studies in mice have suggested that the reduction in intestinal integrity precedes hepatic injury in MASLD, as disruption of the intestinal epithelial barrier and the GVB occurs before hepatic steatosis develops (15). The increase in intestinal permeability and the ensuing liver injury in MASLD are recognized to be due to nutrient and microbial effects on the gut epithelium, microbial translocation, and immune dysregulation.

### Nutrient effects on the intestinal barrier.

Several nutrients have been linked to impairing the intestinal epithelial barrier (16). Fructose is particularly relevant to MASLD pathogenesis due to its association with MASLD risk in humans when consumed in excess (17). Experimental studies in mice and in vitro reported mixed results regarding fructose’s impact on the intestinal epithelial barrier. One study indicated that glucose, not fructose, damages the barrier (18), while others suggested that fructose severely impairs barrier integrity, possibly by direct effects on tight junction proteins (19–21). Although no studies have specifically examined fructose’s effect on barrier function in MASLD patients, fructose, unlike glucose, was associated with a compromised barrier in healthy adults (22), but did not cause the same injury in a recent small, double-blind crossover pilot study of ten individuals with obesity (23). In short, excess carbohydrate consumption can significantly disrupt the intestinal barrier and should be avoided (24).

Lipids also affect the intestinal epithelial barrier. Polyunsaturated fats generally protect the intestinal barrier, (25) whereas multiple experimental models show that dietary saturated and n-6 polyunsaturated fatty acids can harm the intestinal epithelial barrier. For example, in an enterocyte in vitro model, treatment with the saturated fatty acids palmitate (C16:0) and stearate (C18:0) increased epithelial permeability, as measured by transepithelial electrical resistance (26). Compared with mice fed a low-fat diet, mice fed diets high in saturated fat or n-6 polyunsaturated fat had higher colonic permeability (27). Cholesterol oxidation products such as oxysterols also injure the intestinal barrier through a reduction of tight junction proteins (28).

### Microbes affect intestinal barrier, translocation, and immune dysregulation.

Gut dysbiosis is the imbalance between beneficial and harmful gut microorganisms. In patients with MASLD, dysbiosis can result from increased intestinal microbial load, reduced microbial diversity, and/or a dominance of pathogenic microbes. These pathogenic microbes can decrease expression of tight junction and GVB junction proteins (15), secrete proteases to degrade these proteins (29), trigger local intestinal inflammation (30), and damage or invade the protective mucosal layer (31).

**Figure 2:**
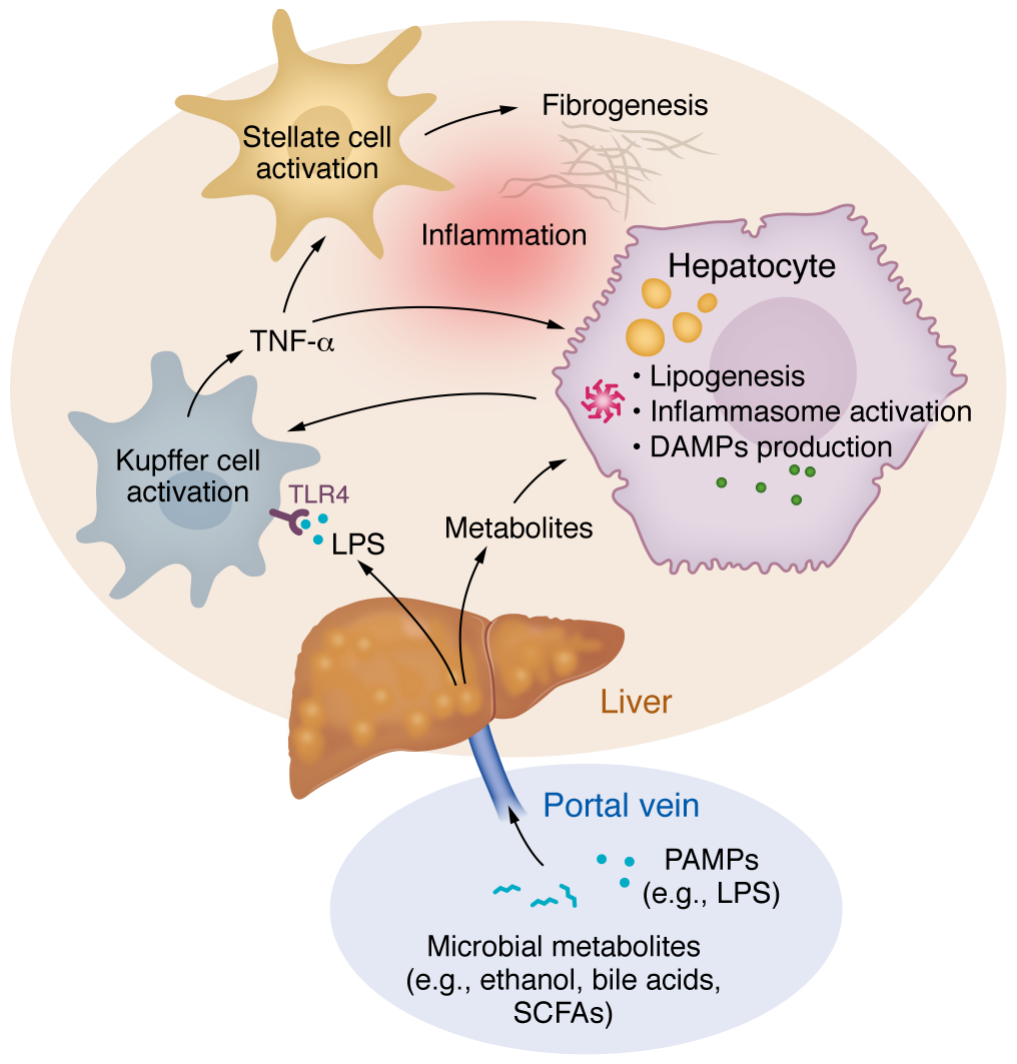
Microbial products and metabolites influence fibrogenic responses in the liver. PAMPs (such as LPS) and microbial metabolites (e.g., SCFAs, ethanol, bile acids) that translocate to circulation from the gut enter the liver via the portal vein. LPS stimulates a proinflammatory and ultimately fibrogenic cascade via Kupffer cells, which are liver-resident macrophages. Gut dysbiosis alters production of microbial metabolites, and these irregularities are associated with a variety of adverse effects in hepatocytes, including inflammatory processes that exacerbate fibrogenesis.

Disruption of the intestinal epithelial and vascular barrier leads to the translocation of gut microbiota and gut microbial components from the intestinal lumen into the systemic and portal circulations (Figure 1). Bacterial wall products such as lipopolysaccharide (LPS, endotoxin) enter the bloodstream, triggering steatotic and inflammatory responses within the liver. LPS, a pathogen-associated molecular pattern (PAMP), is recognized by the TLR4 receptor on hepatic Kupffer cells, integral to the innate immune response. This recognition activates myeloid differentiation factor 88 (MyD88) and interferon regulatory factor 3 (IRF3), initiating a proinflammatory cascade that results in TNF-α secretion (32). The subsequent liver disease progression from steatosis to steatohepatitis and fibrosis involves Nod-like receptor protein 3 (NLRP3) inflammasome activation and the liver’s production of damage-associated molecular patterns (DAMPs). These processes activate caspase 1, proinflammatory cytokines such as IL-1β, and Kupffer cells, which in turn stimulate hepatic stellate cells’ fibrogenic response (Figure 2). Translocated bacteria can also exert hepatic injury in MASLD through the production of microbial metabolites (discussed in more detail below). Indeed, we have shown that metabolite signatures (versus bacterial taxonomic stratification themselves) may be better able to distinguish noncirrhotic MASH from healthy human controls (33).

In addition to the hepatic immune effects of microbial translocation, translocation also results in adipose tissue inflammation, in part through LPS signaling. LPS targets the aforementioned TLR4 inflammatory signaling in adipocytes, promotes the inflammatory M1 macrophage transition, and prompts programmed adipocyte cell death through the activation of caspase signaling (34–36). The resultant effects on adipokine dysregulation that impair both glucose and lipid homeostasis are, in turn, likely to contribute to MASLD pathogenesis.

Viable microbes that escape the intestinal lumen encounter an additional line of surveillance by the intestinal immune system in the lamina propria and in the mesenteric lymph nodes (37). The exact route of translocation through the epithelial barrier is unclear, but may involve endocytosis or transcytosis (38). Full immune escape is possible, however, as demonstrated by the clinical syndrome of spontaneous bacterial peritonitis where translocated bacteria infect sterile ascitic fluid in patients with cirrhosis and portal hypertension (37). Additionally, there are reports of possible immune escape of viable microbes into the liver itself. Patients with MASH had increased bacterial DNA levels of the class Gammaproteobacteria, family Pseudomonadaceae, and order Pseudomonadales in the liver, while the family Lachnospiraceae was reduced (39). In another study of 26 human liver biopsies, 16s rRNA sequencing identified bacterial species in the liver with a predominance of Proteobacteria and a relative lower abundance of Firmicutes (40). 16s rRNA sequencing was also performed in livers of 82 patients with varying degrees of fibrosis and it was found that the liver bacteria comprised mostly Proteobacteria (41). Bacteria have also been observed in mouse livers, although at significantly lower abundance than in the intestines. Isolated bacteria were successfully cultured and visualized using 16S FISH and transmission electron microscopy, which showed bacteria in hepatic sinusoids and being engulfed by resident Kupffer cells (40). Validation of these studies in humans is challenged by the difficulty of obtaining sterile liver biopsy tissue and reliance on 16s rRNA sequencing, a surrogate measure of bacterial burden. Once validated, however, these studies would shift the paradigm of the liver being a sterile organ (42) and help provide a direct link between intestinal barrier disruption and subsequent liver inflammation in MASLD.

In humans with MASLD, there is evidence that microbial translocation and subsequent immune dysregulation occur. Plasma LPS levels, as a surrogate maker for increased intestinal permeability, are significantly elevated in MASH patients compared with patients with MASLD, as are hepatic TLR4 mRNA levels. A similar, albeit nonstatistically significant trend, is observed with MyD88 hepatic mRNA levels (43). Recently, in biopsy-proven MASH patients, researchers observed higher serum LPS and hepatocyte LPS compared with controls. These levels correlated with serum ZO, an aforementioned marker of increased gut permeability. There were also higher amounts of TLR4^+^ macrophages in MASH patients as compared with control and patients with only steatosis; and these levels correlated with serum LPS (44). Finally, hepatic NLRP3 levels are increased in MASH patients compared with early stage MASLD patients (45).

These mechanisms collectively lead to gut dysbiosis, compromised intestinal barriers, and liver injury in MASLD. Below, we will examine the specific bacterial, viral, and fungal organisms and metabolites involved in human MASLD pathogenesis.

## Microbiota implicated in human MASLD

### Bacterial microbiome.

Small intestinal bacterial overgrowth (SIBO) is a quantitative increase in bacteria in the small intestine. It can be assessed by direct culturing of small intestinal contents or different breath tests. Experimental SIBO can lead to liver injury and inflammation even in the absence of a direct liver toxin. SIBO is prevalent in patients with MASLD, and up to 35% of patients show SIBO (46). SIBO is more prevalent in patients with MASH cirrhosis (47.1%) compared with patients with MASLD (47). *Escherichia coli* and *Staphylococcus aureus* were the most commonly cultured bacterial organisms from small bowel aspirates (47).

Numerous studies have assessed the bacterial composition in patients with MASLD and MASH. However, many of these studies have small sample sizes, lack validation cohorts, fail to control for metabolic comorbidities, or do not carefully characterize liver disease activity. Additionally, results from sequencing studies are influenced by factors such as DNA extraction methods, the use of different sequencing regions, sequencing depth, and methods of analysis. Consequently, there is considerable heterogeneity in the reported changes in the bacterial gut microbiota in patients with MASLD.

Reduced bacterial diversity has been consistently reported in multiple studies in patients with MASLD as compared with healthy controls (48, 49). Using a longitudinal cohort of 867 subjects from China with MASLD and 679 controls, an increased abundance of the fecal microbiota phylum Fusobacteria and family Veillonellaceae were associated with increased MASLD risk, whereas families Rikenellaceae and Barnesiellaceae, and strain *Bifidobacterium adolescentis* were associated with a lower presence of MASLD as assessed by 16S rRNA gene sequencing (50). Some of these changes can be used to monitor response to treatment. For example, the abundance of bile-acid–sensitive *Veillonella* increases in patients responding to an FGF-19–mediated suppression of bile acid synthesis (51).

A metaanalysis of 54 studies with 8,894 subjects showed reduced prevalence of antiinflammatory microbes (family, Ruminococcaceae; genera, *Alistipes*, *Blautia*, and *Faecalibacterium*) and higher abundance of potentially inflammatory microbes (family, Fusobacteriaceae and Enterococcaceae; family, Escherichia and Granulicatella) in patients with MASLD (49). Another metaanalysis and systematic review of 28 published studies (3,566 subjects with confirmed MASLD) confirmed reduced Shannon diversity and a decrease in the relative abundance of short chain fatty acid–producing (SCFA-producing) bacteria such as *Ruminococcus*, *Faecalibacterium*, and *Coprococcus*. This review also showed an increase in *Escherichia* in individuals with MASLD (52). These results were overall confirmed in a metaanalysis including 577 patients with MASLD and 688 controls (53). The relative abundance of *Escherichia*, *Prevotella*, and *Streptococcus* was increased, while *Coprococcus*, *Faecalibacterium*, and *Ruminococcus* were decreased (53, 54).

Beyond distinguishing patients with MASLD and controls, several studies characterized fecal microbiota features in patients with progressive MASLD and those developing fibrosis. There is a stepwise increase of *E*. *coli* and a stepwise decrease of *Eubacterium rectale*, *Eubacterium eligens*, *Facelibacterium prausnitzii*, and *Dorea longicatena* as patients progress from MASLD steatosis to MASLD with mild fibrosis (F1/2/3) and eventually to MASLD cirrhosis (F4) as assessed by metagenomic sequencing (55, 56). Interestingly, using 16S rRNA gene sequencing, Ruminococcaceae decrease and Veillonellaceae increase as fibrosis became more severe in nonobese, but not obese patients with MASLD (57). Using culturomics, four bacterial strains (*Enterococcus faecium*, *Streptococcus oralis*, *Escherichia coli*, and *Klebsiella pneumoniae*) were isolated from children with MASLD and shown to have a lipogenic effect in HepG2 cells. A larger cohort analysis (*n* = 161) using metagenomic sequencing confirmed that these identified bacterial strains were stepwise enriched from obese to MASLD and MASH children (58). Taken together, the transition from a healthy to a steatotic liver is associated with SIBO, a decrease of beneficial bacteria, and an increase of pathobionts in feces.

### Mycobiome.

Beyond bacteria, the fungal microbiota has been characterized in patients with MASLD. Similar to the bacterial microbiota, fungal diversity is also decreased in fecal samples of MASLD subjects (*n* = 21) as compared with controls (*n* = 20) (59). *Saccharomyces cerevisiae* and *Schizosaccharomyces pombe* were more abundant in feces of the healthy group, while higher proportions of *Mucor ambiguous* were found in feces and saliva of subjects with MASLD (59). Patients with MASH or advanced fibrosis had a distinct fecal mycobiome as compared with simple steatosis or minimal fibrosis, which was characterized by increased *C*. *albicans*, *Babjeviella inositovora*, *Mucor sp.*, unknown *Hanseniaspora*, unknown *Pleosporales*, and *Pichia barkeri* (60). Notably, these changes were observed in nonobese patients with MASLD for which risk factors are not well understood. The importance of the mycobiome in MASLD pathogenesis was further highlighted in a microbiota-humanized mouse model. In this model, oral antifungal treatment with amphotericin B reduced steatohepatitis and liver fibrosis induced by a Western diet (60). However, the mechanism by which fungi contribute to MASLD remains unknown. Patients with MASLD and advanced liver fibrosis had higher systemic anti–*C*. *albicans* IgG levels than controls or patients with minimal fibrosis (60), indicating that fungi or their products could translocate from the gut to the liver through a compromised gut barrier.

### Virome.

In a study of fecal viromes from patients with MASLD and controls, histologic markers of MASLD severity were associated with reduced viral diversity and a lower proportion of bacteriophages (phages) compared with other intestinal viruses (61). Specific gut viral taxa, such as *Lactococcus* phages, were decreased in patients with advanced fibrosis (61). Interestingly, consumption of low to moderate amounts of alcohol in MASLD patients accounted for differences in the intestinal viromes. For example, viral diversity of the alcohol-consuming MASLD population was similar to those of patients with alcohol use disorder and significantly higher than the non–alcohol-consuming MASLD population or controls (62). Mechanistic studies are needed to investigate a potential causal role of gut virome changes in disease progression.

**Figure 3:**
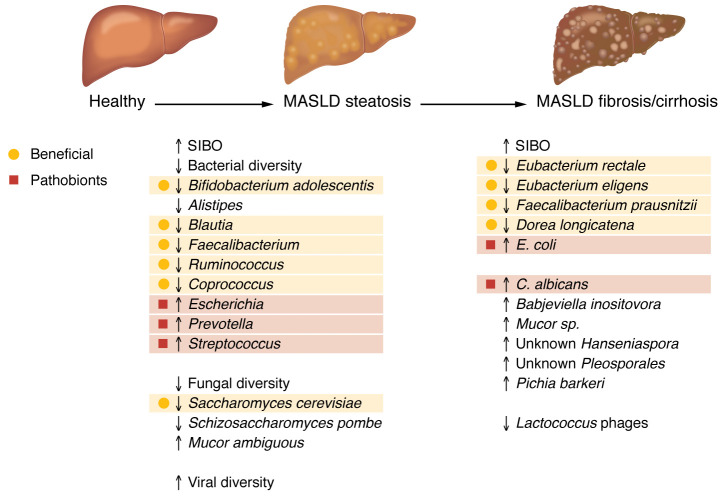
Changes in the gut microbiota associated with MASLD. Patients with steatosis exhibit alterations in their gut bacterial, fungal, and viral microbiomes. As the disease progresses towards fibrosis and cirrhosis, further microbiota changes are observed.

A summary of the above microbiome changes is depicted in Figure 3.

## Microbial metabolites in MASLD pathogenesis

**Figure 4:**
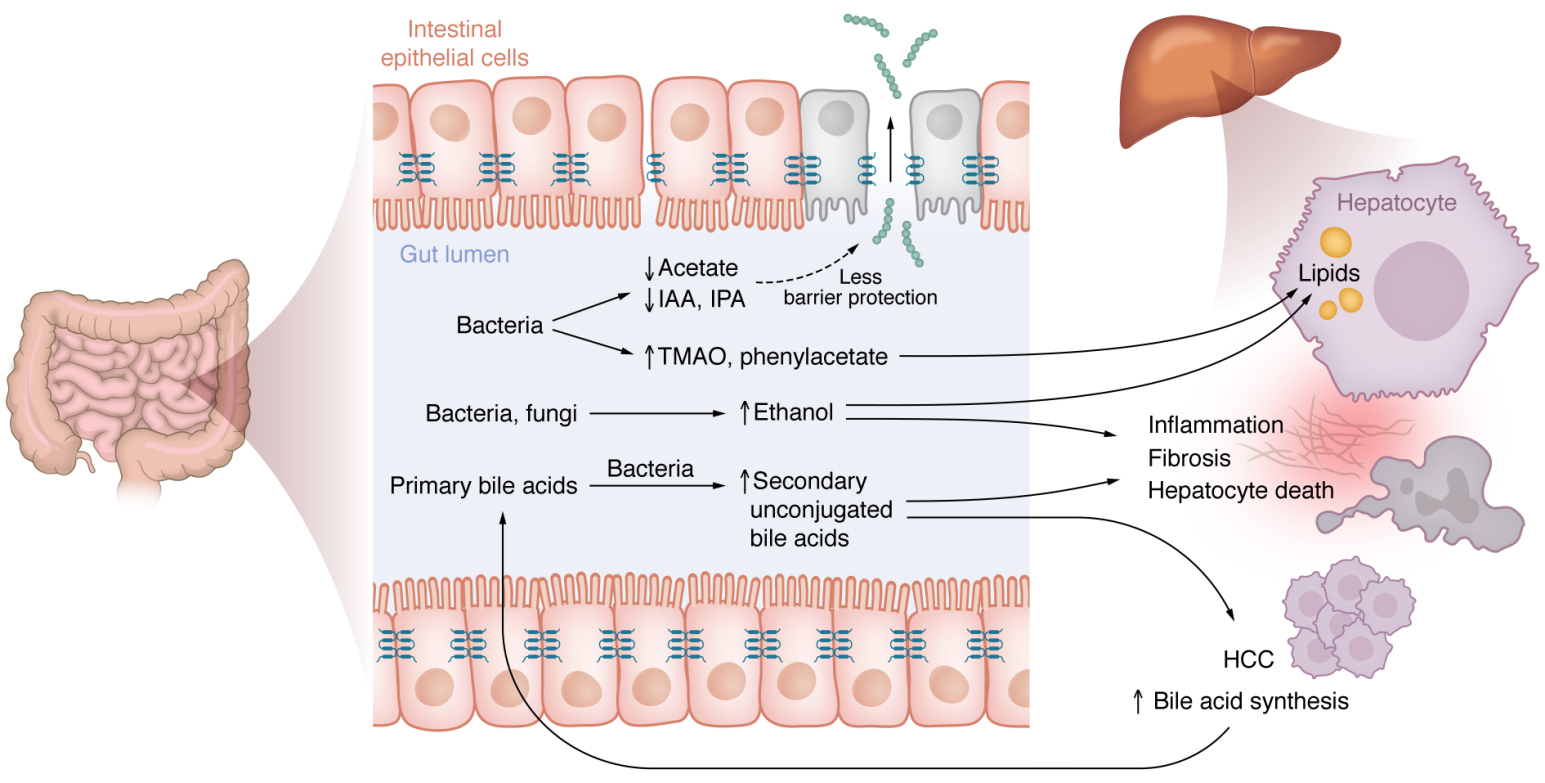
Influence of gut microbial metabolites on MASLD. A dysbiotic gut microbiota produces less beneficial metabolites such as the SCFA acetate, or tryptophan metabolites indole-3-propionic acid (IPA) and indole-3-acetic (IAA), which stabilize the gut barrier during health. On the other hand, ethanol as a fermentation byproduct of bacteria and fungi causes triglyceride accumulation and death of hepatocytes, resulting in inflammation and fibrosis. Patients with MASLD have an increased bacterial metabolism of primary to unconjugated secondary bile acids in the gut, which are hepatotoxic and cause disease progression and HCC. It might also result in increased bile acid synthesis by hepatocytes.

The gut microbiota play an important role in the development and progression of MASLD by influencing the host’s metabolic environment. Gut microbes produce or metabolize a variety of metabolites, such as ethanol, SCFAs, and bile acids, which can affect liver function and overall metabolic health. Alterations in the composition and activity of the gut microbiota can lead to dysregulated production of these microbial-derived metabolites, contributing to inflammation, insulin resistance, lipid accumulation in the liver, and hepatic fibrosis. Understanding these interactions offers potential therapeutic targets for MASLD (Figure 4).

### Ethanol.

Ethanol is a fermentation byproduct of bacteria and fungi, naturally produced in the human body. It is typically metabolized by enzymes in the intestinal epithelial cells or after being absorbed into the bloodstream and transported to the liver via the portal vein (63). Disruptions in gut homeostasis can lead to elevated ethanol production by intestinal microbes. Studies indicate that patients with MASH exhibit higher levels of peripheral blood alcohol, with even higher concentrations found in the portal vein, especially as liver disease severity increases (64–66). In extreme cases, excessive microbial ethanol production can overwhelm the liver’s metabolic capacity, leading to high blood alcohol levels and a rare condition known as autobrewery syndrome (67). Research involving the transplantation of stool from a MASH patient with ethanol-producing *Klebsiella pneumoniae* into mice has demonstrated that this can cause steatohepatitis, a process that can be mitigated by using phages targeted against the ethanol-producing bacteria (68). Other intestinal bacteria also produce ethanol and contribute to liver inflammation (69). This evidence underscores a causal link between ethanol-producing bacteria and the development of steatohepatitis.

### Bile acids.

Bile acids, primary metabolites synthesized by hepatocytes from cholesterol, play essential roles in food digestion within the small bowel and are reabsorbed in the terminal ileum. Beyond digestion, bile acids interact with receptors like Farnesoid X receptors (FXR) and TGR5, profoundly influencing glucose and lipid metabolism and regulating their own synthesis through the secretion of FGF-19 in the terminal ileum. Bile acids also modulate the gut microbiota and are metabolized by these microbes in turn. Thus, disturbances in any of the factors that change bile acids can have profound effects on metabolic diseases. Bile acid homeostasis is disturbed in patients with MASLD. There is heterogeneity in results reporting changes in bile acid associated with MASLD, which may result from different analytical methods, small sample size, and confounding factors such as medications, age, BMI, and sex (33, 70).

Although serum bile acid levels do not significantly differ in patients with MASLD compared with controls, several studies found serum bile acids associated with greater liver disease severity in patients with MASLD. Secondary unconjugated bile acids, particularly lithocholic acid species, were increased in patients with mild and significant fibrosis in a Chinese cohort of patients with MASLD (*n* = 550) (71). Similar results were found in a Hispanic cohort (*n* = 390), where total and primary conjugated bile acids were higher in subjects with fibrosis, with high lithocholic acid levels strongly associated with advanced fibrosis (72). Additionally, Caussy et al. noted an increase in total bile acids and primary-conjugated bile-acid proportion with increasing liver fibrosis stage in patients with MASLD (*n* = 312) (73). Plasma bile acids, FGF19, or de novo bile acid synthesis (measured as serum C4 levels) did not correlate with fibrosis stage within a noncirrhotic cohort of patients with MASH after adjusting for BMI and age (*n* = 279) (33) demonstrating the role metabolic risk factor confounding may have on the interpretation of MASLD microbiome studies.

Increased systemic levels of secondary bile acids such as deoxycholic acid and lithocholic acid may exert direct hepatotoxic effects, potentially contributing to liver damage (74). These hydrophobic bile acids are particularly effective in activating the TGR5 receptor (75), which may lead to decreased activity of FXR and subsequent increase of de novo bile acid synthesis as described in patients with MASH compared with healthy controls (76). The therapeutic potential of restoring this pathway is supported by the successful use of the semisynthetic FXR agonist obeticholic acid, which has antifibrotic effects in patients with MASH (77). Additionally, microbial-derived deoxycholate has been implicated in the pathogenesis of HCC, underscoring the complex role of gut microbiota in liver disease (78, 79).

Recently, new amino acid or amine-conjugated bile acids (bile acid amidates) have been identified (80, 81). One of these bile acids is 3-succinylated cholic acid, which is synthesized by *Bacteroides uniformis* and was found to be lower in stool from patients with MASLD compared with controls. Supplementation of this lumen-restricted 3-succinylated cholic acid alleviated steatohepatitis by expanding the population of *Akkermansia muciniphila* in mice (82). Other secondary bile acids induce Tregs, either directly or by stimulating dendritic cells, which inhibit Th17 cells in the intestine (83–85). It will be interesting to investigate whether these secondary bile acids suppress intestinal inflammation in patients with MASLD, which is closely linked to intestinal barrier dysfunction (86).

### SCFAs and branched-chain fatty acids.

SCFAs are key microbial fermentation products of nondigestible proteins and fibers, which play crucial roles in immune regulation and maintaining gut barrier integrity. Several SCFA-producing bacteria are depleted in the microbiota of patients with MASLD. Consequently, plasma levels of acetate are generally lower in MASLD patients (*n* = 100) compared with healthy controls (*n* = 50), while other SCFAs like propionate are elevated and linked to liver fibrosis (87). Propionate, in particular, is associated with increased fibrosis and may also contribute to insulin resistance in humans (87, 88). Conversely, acetate produced by *Bifidobacterium pseudolongum* protects against diet-induced steatohepatitis and MASH-associated HCC by stabilizing the gut barrier and exerting a direct antiproliferative effect on liver cells (89). Several other metabolites are also produced as fermentation products of proteins such as branched-chain fatty acids (BCFA), which include iso-butyric acid and iso-valeric acid. The production of BCFAs is impacted by diet quality, and fecal BCFA levels have been correlated with less consumption of dietary insoluble fiber (90). Patients with MASLD and MASH have higher total BCFAs in the liver as compared with controls. Total hepatic BCFAs correlated with disease severity in patients with MASLD (91). Further research is needed to determine whether hepatic BCFAs originate from the microbiota and whether they play a role in the pathogenesis of MASLD.

### Other microbial metabolites affecting MASLD.

Other microbial-derived metabolites have also been implicated in the pathogenesis of MASLD. MASLD patients have higher circulating levels of the microbial metabolite trimethylamine *N*-oxide (TMAO) as compared with controls (92). TMAO promotes lipid accumulation in cultured cells (93). Serum phenylacetate was associated with steatosis in women with obesity, and phenylacetate promoted hepatic triglyceride accumulation in mice (94).

On the other hand, other metabolites produced by the gut microbiota might be beneficial for MASLD. Pentadecanoic acid produced by *Parabacteroides distasonis* is protective against diet-induced steatohepatitis in mice (95). Fecal levels of the tryptophan metabolites indole-3-propionic acid and indole-3-acetic acid were lower in patients with MASLD compared with healthy controls. Indole-3-propionic acid and indole-3-acetic acid administration ameliorated diet-induced steatohepatitis in mice by stabilizing the gut barrier and through direct effects on the liver (96, 97).

## Therapeutic modulation of the microbiome in MASLD

In the current era of gut-microbiome discovery in MASLD, efforts are underway to translate findings on gut microbial and metabolite changes into patient care (98). Still, clinical trials evaluating microbiome-based therapies in MASLD are limited but are, fortunately, increasing in number. Recent studies suggest that validated interventions such as nutrition, exercise, and bariatric surgery favorably impact the microbiome, which may contribute to the efficacy in MASLD. Emerging research explores targeted microbiome-based therapies using prebiotic, probiotic, and postbiotic approaches to improve treatment outcomes (99–101) (Table 1).

### Standard of care.

The current standard of care in MASLD includes dietary and exercise recommendations and medications. Of the diets, the Mediterranean-style diet is often recommended (1). This diet increases microbial diversity in part due to bioactive components like fiber, polyphenols, unsaturated fatty acids, and fermentation products (102–105). These bioactives, directly and via conversion to active metabolites, support microbial and mitochondrial health, potentially reducing hepatic fat, inflammation, and fibrosis (106). Additionally, the Mediterranean diet is low in red meat, sugar, salt, additives, saturated fat, and choline, which may further benefit microbial diversity and function (92, 107–110). Interestingly, vitamin E, regular exercise, and even bariatric surgery have been shown to improve microbial diversity (111–113), a mechanism that warrants further exploration.

While there is only one FDA-approved medication for MASLD for patients with at least moderate fibrosis (114), many patients have been prescribed metformin or statins to manage concomitant insulin resistance/diabetes or hyperlipidemia, respectively. Metformin and statins have important but seemingly opposing effects on the gut microbiome. Statins may, in fact, worsen glucose homeostasis through disruptions of the gut microbiome (115), while metformin’s effects on the gut microbiome improve glucose homeostasis. In a randomized, double-blind trial involving treatment-naive type 2 diabetes patients and controls, metformin modified the gut microbiota, and transplanting feces from metformin-treated individuals into germ-free mice improved glucose control without affecting body weight or fat mass (116). These studies demonstrate that there can be multiple factors that impact the gut microbiome in MASLD.

### Prebiotics and food.

Prebiotics, like lactulose and the bioactive fibers and polyphenols in plant-based foods, promote the growth of beneficial bacteria and their metabolites, potentially improving liver health in MASLD patients (117–119). For example, a randomized controlled trial (RCT) evaluating 27 adults consuming a high-fiber roll twice daily for two months showed a significant reduction in liver steatosis by FibroScan, accompanied by significant changes in microbial metabolites (120). In another RCT, 19 adults taking the prebiotic fiber inulin versus the carbohydrate maltodextrin for 12 weeks saw an increase in *Bifidobacterium* without significant changes in liver fat or function tests (121). The variability in outcomes may relate to specific fiber types, baseline diets, and subject characteristics. Clinical intervention studies with polyphenols favorably impact the microbiome (122, 123), though their evaluation in MASLD is still limited.

### Probiotics and bacteria.

Probiotics, live bacterial products (LBPs), and fecal microbiota transplantation (FMT) are live bacterial interventions being evaluated for MASLD treatment (124–126). Probiotic trials are heterogeneous, typically using combinations of *Lactobacillus* and *Bifidobacterium*. A metaanalysis of 27 studies, including a 12-week RCT of 68 subjects with MASLD, found improvements in liver steatosis, though liver function tests did not improve (127, 128). Recent RCTs with various probiotics, such as lactic acid bacteria combinations and *Bacillus coagulans,* have shown mixed results (129, 130). FMT studies for MASLD are limited to three small RCTs, with varying effects on liver fat and function (131). LBPs, such as those recently approved for *Clostridium difficile* infection, offer the potential benefits of a defined therapeutic microbial consortium, although no trials have been reported to date (132, 133).

### Postbiotics and Metabolites.

Postbiotics, beneficial metabolites produced by gut bacteria (e.g., butyrate, modified polyphenols) or by fermenting foods (e.g., lactic acid, acetate, modified polyphenols), may improve gut health and reduce liver fat, inflammation, and fibrosis (134). A recent trial of 50 subjects randomized to butyrate, vitamin D3, and zinc versus placebo showed a significant improvement in steatosis by the fatty liver index and steatosis index, an effect seen previously in animal model studies for MASLD (135). Modified polyphenols such as urolithin A have only been evaluated in animal models to date, where they have demonstrated a decrease in steatohepatitis (136). Fermented foods have not been evaluated for MASLD, but a recent RCT showed they can significantly increase microbial diversity and decrease systemic inflammation (137).

While research on microbiome-targeting therapies for MASLD is still in its early stages, some findings suggest these interventions may complement standard care. Investigating individual components like dietary bioactives, microbial species, and metabolites may yield valuable insights, underscoring the importance of whole-food diets and leading to new targeted therapies. Postbiotics and fermented foods, which may bypass the need for a healthy microbiome by directly supplying beneficial metabolites, remain particularly underexplored and hold promise as potential new treatments for MASLD. Further research into the small intestine microbiome and metabolites with newly available tools may also provide new therapeutic avenues (138).

## Conclusion

Gut dysbiosis is pivotal in MASLD pathogenesis, with microbial imbalance being associated with both the onset and progression of liver disease. Understanding these associations supports the development of microbial-based therapies to restore gut balance. Further research is needed to refine these therapies for personalized treatment of steatosis, inflammation, and fibrosis in MASLD. This includes investigating xenobiotic metabolism to predict therapy responders and nonresponders.

## Figures and Tables

**Table 1:**
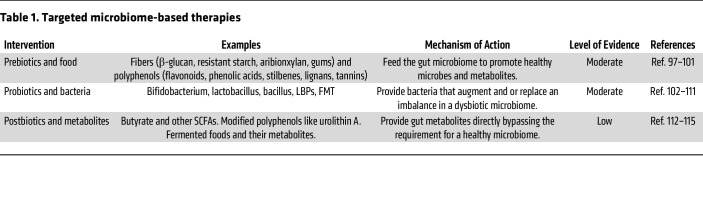
Targeted microbiome-based therapies

## Version 1 04/01/2025

Electronic publication
